# Probing of multiple magnetic responses in magnetic inductors using atomic force microscopy

**DOI:** 10.1038/srep20794

**Published:** 2016-02-08

**Authors:** Seongjae Park, Hosung Seo, Daehee Seol, Young-Hwan Yoon, Mi Yang Kim, Yunseok Kim

**Affiliations:** 1 School of Advanced Materials Science and Engineering, Sungkyunkwan University (SKKU), Suwon 440-746, Republic of Korea; 2 Fundamental Technology Group, Central R&D Institute, Samsung Electro-Mechanics Co., Suwon 443-743, Republic of Korea

## Abstract

Even though nanoscale analysis of magnetic properties is of significant interest, probing methods are relatively less developed compared to the significance of the technique, which has multiple potential applications. Here, we demonstrate an approach for probing various magnetic properties associated with eddy current, coil current and magnetic domains in magnetic inductors using multidimensional magnetic force microscopy (MMFM). The MMFM images provide combined magnetic responses from the three different origins, however, each contribution to the MMFM response can be differentiated through analysis based on the bias dependence of the response. In particular, the bias dependent MMFM images show locally different eddy current behavior with values dependent on the type of materials that comprise the MI. This approach for probing magnetic responses can be further extended to the analysis of local physical features.

Magnetic materials have been broadly explored for multiple applications including in inductors, transformers, electric motors and generators^1^,^2^,^3^. Furthermore, multiferroic materials, which possess more than one ferroic order parameter, *e.g.* magnetic properties, are of great interest due to their interesting physical properties and potential applications^4^,^5^,^6^. Many of these applications are underpinned by the existence of eddy current. The eddy current is a circular electrical current induced within conductors by changes in the magnetic field due to Faraday’s law of induction and the direction of the eddy current is opposite that of the coil current which generates a dynamic magnetic field^7^. Accordingly, since the eddy current reduces the magnitude of the original magnetic field, the eddy current can be a source of energy loss in magnetic applications^1^,^2^,^3^. Hence, investigating the magnetic energy loss is a key first step towards significant performance improvement. Thus, to fully understand the magnetic behavior in these practical applications, it is necessary to explore the fundamental magnetic behavior of various magnetic properties at the nanoscale as well as to understand the operational mechanisms in real device structures.

Previously suggested atomic force microscope (AFM) based approaches, such as eddy current microscopy (ECM)^8^,^9^,^10^ and magnetic force microscopy (MFM)^11^,^12^, only provide information on individual magnetic properties such as eddy current and magnetic domains for ECM and MFM, respectively. Furthermore, there was a recent report on piezomagnetic force microscopy, which provides magnetostrictive strain induced by a coil current^13^. However, these approaches are limited for simultaneously exploring multiple types of magnetic properties at the nanoscale.

If multiple types of material properties can be simultaneously monitored through analysis of the cantilever dynamics, sufficient information may be obtained to fully understand the fundamental magnetic behavior of the materials as well as the operational mechanisms of real device structures^12^,^14^,^15^,^16^.

Here, we demonstrate probing of the multiple magnetic responses associated with eddy current, coil current and magnetic domains in model magnetic inductor (MI) samples using multidimensional magnetic force microscopy (MMFM). We have chosen commercially available real devices, *i.e.* MIs, as model systems because they are ideal for showing the feasibility of MMFM as their basic material properties are well known. Furthermore, since eddy current loss is of major importance in MI samples, the probing of the multiple magnetic responses is significant for further practical improvement of these real devices.

## Results and Discussion

**Figure 1:**
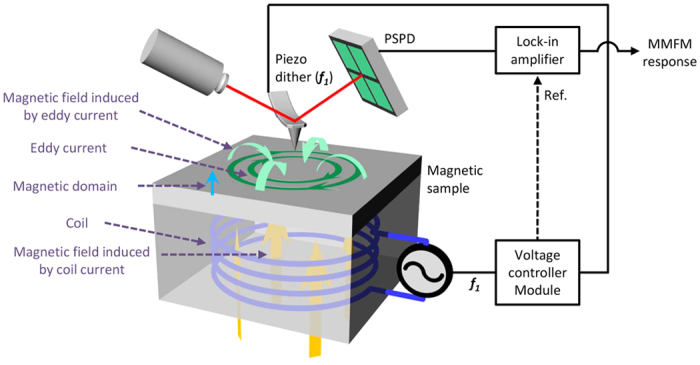
Schematic diagram of MMFM set-up.

Figure 1 shows a schematic diagram of a MMFM setup for exploring the magnetic response associated with the eddy current, coil current and magnetic domains. According to Ampère’s circuital law, a dynamic magnetic field can be generated by applying ac voltage to the coil as it induces current flow (see Supplementary Figure S2). At the same time, the dynamic magnetic field generates circular current, *i.e.* eddy current, within the MI due to periodic changes in the magnetic field^9^. Accordingly, the eddy current, with a direction opposite that of the current flow through the coil, induces an additional magnetic field according to Lenz’s law. On the other hand, since soft magnetic particles are included in the MIs, the magnetic domains can contribute to the magnetic response as well. It is worth noting that the interaction between the static magnetic domain and the magnetized tip is typically measured by detecting changes in the amplitude or phase of the oscillation of the cantilever. This indicates that the static magnetic domains can contribute to the dynamic magnetic response. Hence, the dynamic magnetic response can be basically induced by three different origins, *i.e.* the static magnetic domain and the dynamic magnetic fields induced by the coil and eddy currents.

During the operation of MMFM, since the dynamic magnetic fields generated by coil and eddy currents can vibrate the cantilever at the frequency of the ac voltage applied through the coil^17^, we apply ac voltage to the coil at the vicinity of its resonance frequency. At the same time, the same frequency, which is synchronized with the ac voltage applied to the coil, is applied to the piezo dither to mechanically vibrate the cantilever (see details for the use of the piezo dither in Supplementary Figure S4). We note that operating near the resonance frequency can maximize the response and reduce the relative noise level^18^. In our approach, the total force, *F*_*total*_, acting on the cantilever is written as *F*_*total*_ = *F*_*electrostatic*_ + *F*_*sample*_ + *F*_*coil*_ + *F*_*eddy*_ + *F*_*piezo*_ where *F*_*electrostatic*_, *F*_*sample*_, *F*_*coil*_, *F*_*eddy*_, and *F*_*piezo*_ are forces induced by the electrostatic component, magnetic domains in the sample, the dynamic magnetic field induced by the coil current, the dynamic magnetic field induced by the eddy current, and that resulting from the piezo dither, respectively. There can be an electrostatic component acting between the tip and the sample as ac voltage is applied in the MMFM setup^19^. However, since the two ac voltages are applied separately to the coil and to the piezo dither, it is not a typical vertical component and is expected to be relatively small. Furthermore, since the physical distance between the tip and coil is fairly large (Supplementary Figure S1), the effect is expected to be relatively small as well. It is worth noting that capacitive coupling, which is a common problem in Kelvin probe force microscopy^20^, is also expected to be fairly small for the same reason in spite of the fact that the frequencies of the ac voltage and the vibration of the tip are the same. Indeed, there is no significant change in the electrostatic response from increasing the ac voltage (see details in Supplementary Figure S7). Thus, the electrostatic contribution, *F*_*electrostatic*_, can be negligible. Under several assumptions, each magnetic force acting on the cantilever in the *z* direction, which contains *F*_*sample*_, *F*_*coil*_, and *F*_*eddy*_ can be written as follows


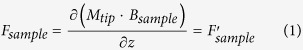



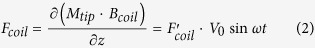



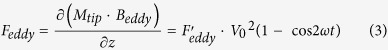


where *M*_*tip*_ and *B* are the tip magnetization and the magnetic field associated with each component, respectively (see details in Supplementary information). The interaction between the total magnetic force and the magnetized tip induces the MMFM response, and it can be measured by detecting changes in the first harmonic oscillation amplitude of the cantilever using a lock-in amplifier. However, it is not possible to distinguish each contribution to the total magnetic response solely from a single MMFM image because the MMFM response is derived from the total change in the cantilever oscillation caused by the three different factors.

**Figure 2:**
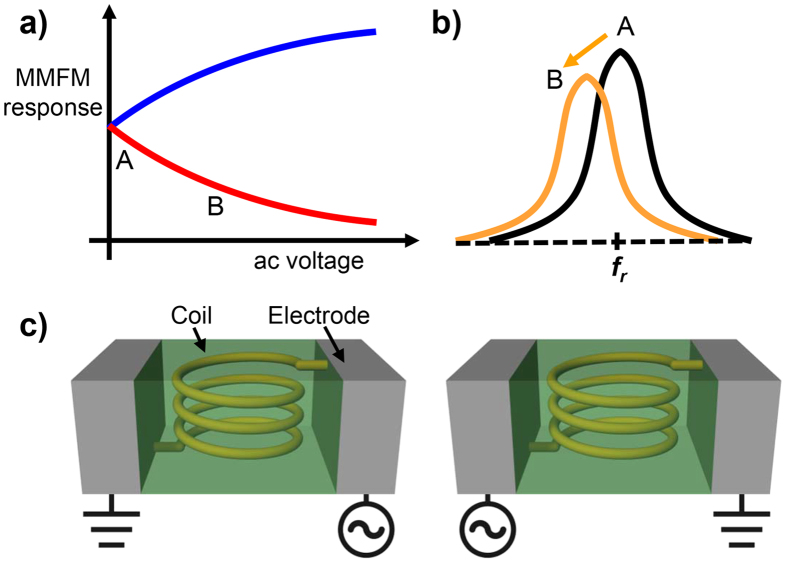
(**a**) MMFM response as a function of ac voltage and (**b**) corresponding change in the MMFM response near the resonance frequency in a frequency domain. (**c**) A configuration of the MI for the application of ac voltage to the coil: the direction of coil current is arbitrarily defined as leftward (left-side) and rightward (right-side), respectively. We note that the MMFM response as a function of ac voltage in Fig. 2(a) depends on the experimental conditions, *e.g.* the direction of the coil current and the magnetization in the tip.

In order to distinguish the contribution of each component to the MMFM response, we monitored the MMFM response as a function of ac voltage based on two aspects: 1) the magnetic force exerted on the cantilever related to the static magnetic domain is independent of the ac voltage as it is an inherent property of the sample and 2) the force associated with the magnetic fields generated by the coil and eddy currents shows linear and nonlinear relationships with the ac voltage, respectively. It was previously reported that the magnetic force resulting from the eddy current is proportional to the square of the coil current^21^,^22^. Consequently, it is expected that the MMFM response exhibits nonlinear behavior when there is sufficient contribution from the eddy current as shown in Fig. 2(a). In addition to the contribution of the eddy current, there can be nonlinear dependence stemming from hysteresis behavior by the soft magnetic particles in response to the external magnetic field generated by the applied ac voltage. However, since the magnetic field induced by the applied *V*_*ac*_ is much smaller than the coercivity of typical soft magnetic particles in MI, these nonlinear portions can be negligibly small^23^. Thus, we can consider that *F*_*eddy*_ dominantly contributes to the nonlinearity of the MMFM response. Accordingly, *F*_*sample*_, *F*_*coil*_, and *F*_*eddy*_ can contribute to the MMFM response with offset, linear, and nonlinear contributions, respectively. It is worth noting that the MMFM response as a function of ac voltage is dependent on the experimental conditions such as the directions of the coil current and the magnetization in the tip. For instance, if the directions of the coil current and the magnetization in the tip are rightward and downward, respectively, the MMFM response follows the red solid line in Fig. 2(a) and the corresponding MMFM response in a frequency domain can be obtained as shown in Fig. 2(b). Then, the opposite of the MMFM response, represented as a blue solid line in Fig. 2(a), is obtained by changing the direction of the coil current. On the other hand, the MMFM response at 0 *V*_*ac*_ is constant regardless of the direction of the coil current as shown in Fig. 2(a) because the contribution is solely from static magnetic domains. Thus, it is expected that the MMFM response at 0 *V*_*ac*_ should be identical to the conventional MFM response.

**Figure 3:**
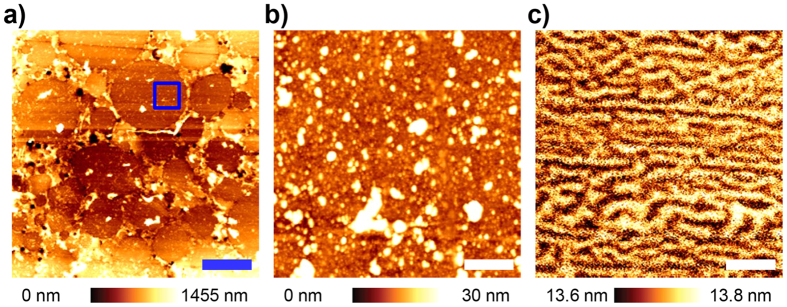
(**a**) Topography image of the polished surface in the MI over an area of 50 μm × 50 μm. (**b**) Topography and (**c**) MFM amplitude images are obtained over an area of 5 μm × 5 μm, corresponding to the blue square in Fig. 3(a). Blue and white scale bars are 10 μm and 1 μm, respectively.

Prior to investigating the MMFM response and each contributing factor, we obtained the topography and conventional MFM amplitude images of the polished surface of the MI. The soft magnetic particles and resin interspersed between the particles are clearly distinguished through the topography as presented in Fig. 3(a). One of the large soft magnetic particles was enlarged over the area corresponding to the blue square in Fig. 3(a). Figure 3(b,c) illustrate the topography and MFM amplitude images obtained in that position, respectively. Consequently, the stripe pattern of magnetic domains within the soft magnetic particle is apparently visible through the MFM amplitude image as shown in Fig. 3(c). This indicates that the magnetic domains exist and are potentially able to contribute to the MMFM response as discussed previously.

**Figure 4:**
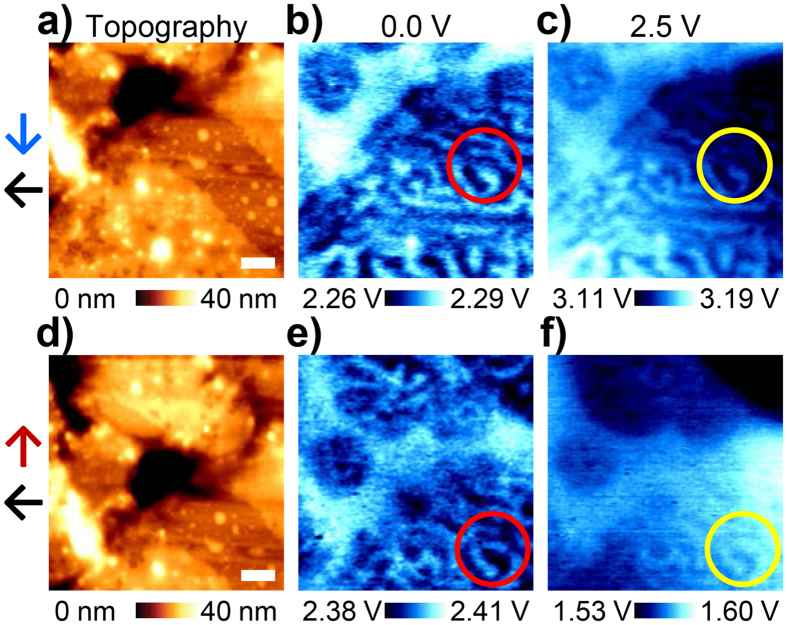
(**a,d**) Topography and corresponding (**b,c,e,f**) MMFM amplitude images with (**b,c**) downward and (**e,f**) upward magnetized tips during the application of (**b,e**) 0 and (**e,f**) 2.5 *V*_*ac*_ to the coil. Blue and red arrows represent downward and upward directions of magnetization in the tip, respectively. Red and yellows circles indicate the same locations on the MI. Note that the direction of the coil current is leftward for both cases as represented by the black arrows. Scale bar is 0.5 μm.

Further, we verified the influence of the experimental conditions on the MMFM response prior to the analysis of each contribution to the MMFM response. The MMFM responses with the magnetized tips, which are in the opposite direction, are obtained in a similar region as can be seen in the two topography images in Fig. 4(a,d). Both the MMFM amplitude images with 0 *V*_*ac*_ in Fig. 4(b,e) show similarities in the stripe pattern. However, the contrast of the magnetic domains is inverted in accordance with the direction of magnetization in the tip (see red circles in Fig. 4(b,e)). This indicates that there is a difference in interaction between the magnetic force resulting from the static magnetic field and the tip with respect to the direction of magnetization in the tip. When ac voltage is applied to the coil, the change in the MMFM response is clearly observed by comparing the MMFM amplitude images at 0 *V*_*ac*_ shown in Fig. 4(b,e) to those at 2.5 *V*_*ac*_ shown in Fig. 4(c,f). As mentioned above, it is verified that the tendency of the MMFM response, which either increases or decreases with the application of ac voltage, changes according to the direction of magnetization in the tip while the direction of the coil current is fixed as leftward. Moreover, the change in the MMFM response under a different coil current direction can be explained in the same manner (see Supplementary Figure S5).

Despite the inversion of contrast in the magnetic domains, the difference in the MMFM amplitude between the two domains, which are represented as bright and dark regions in red and yellow circles in Fig. 4, is nearly constant at approximately 0.02 V under all experimental conditions with the same magnetic tip. These results indicate that the magnetic forces resulting from the static magnetic domains and the coil and eddy currents independently contribute to the MMFM response and that each contribution affects the MMFM response regardless of the experimental conditions. Thus, differentiating each contribution under any experimental condition is valid and feasible.

**Figure 5:**
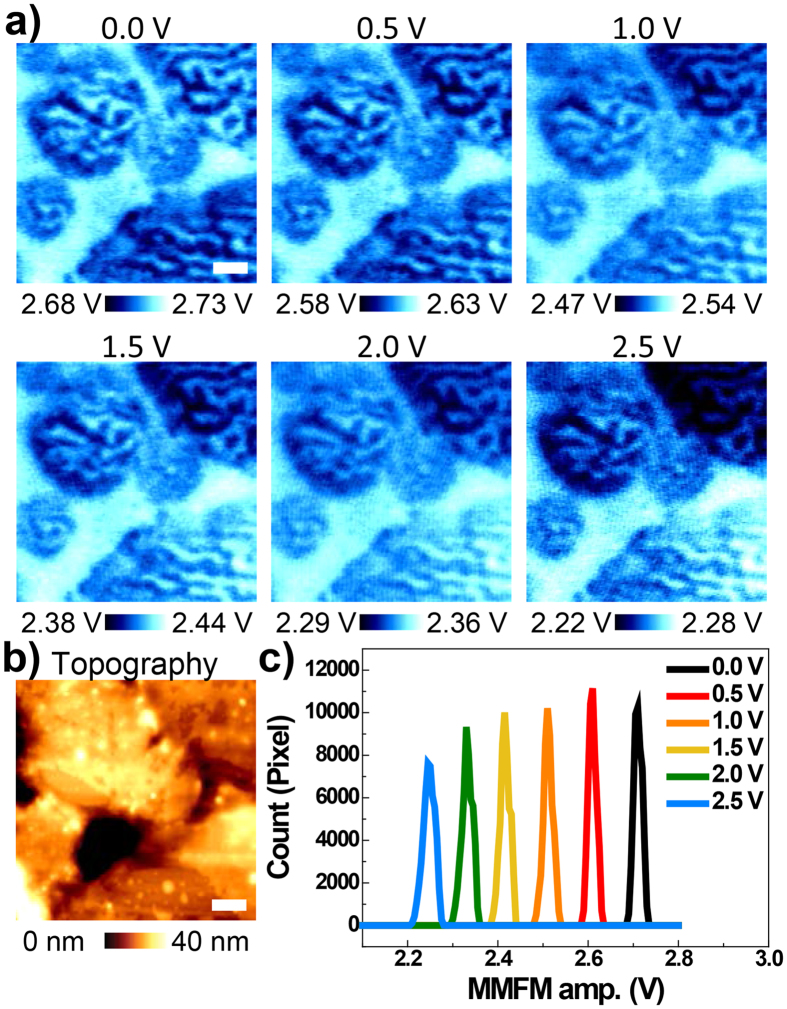
(**a**) MMFM amplitude images as a function of ac voltage to the coil and corresponding (**b**) topography image of the polished surface in the MI over an area of 3.5 μm × 3.5 μm and (**c**) histograms of the MMFM response in Fig. (a). Note that the directions of the coil current and magnetization in the tip are rightward and downward, respectively. Scale bar is 0.5 μm.

In order to explore the dependence of the MMFM response on the applied ac voltage according to each contribution, the MMFM responses with various ac voltages, ranging from 0 to 2.5 *V*_*ac*_, were measured at the same position presented in Fig. 5(a). The obtained MMFM amplitude images show a gradual decrease in the entire MMFM response according to the increase in the applied ac voltage. We note that the experimental conditions are arbitrarily fixed to demonstrate a decrease in the MMFM response with respect to the applied ac voltage as represented by the red solid line in Fig. 2(a). To clearly visualize this progressive decrease in the entire MMFM response, each MMFM amplitude image is represented in the histogram shown in Fig. 5(c). The image clearly shows that the histogram moves towards the left as the applied ac voltage increases. In addition, it seems that the spacing between the peaks of adjacent histograms is constant over the range of the ac voltage. Indeed, the MMFM amplitudes at the peaks in the histograms with respect to 0 to 2.5 *V*_*ac*_ are approximately 2.71, 2.61, 2.51, 2.42, 2.33 and 2.24 V, respectively. Since the differences between those values are around 0.10 V at relatively lower *V*_*ac*_ and decreases as *V*_*ac*_ becomes larger, a linear and nonlinear relationship between the MMFM response and ac voltage, indicating the contribution from the coil and eddy currents, is roughly observed through the histogram as well. However, such analysis based on histograms does not provide sufficient information such as the contribution from the eddy current and relative contributions from each origin to the entire MMFM response.

**Figure 6:**
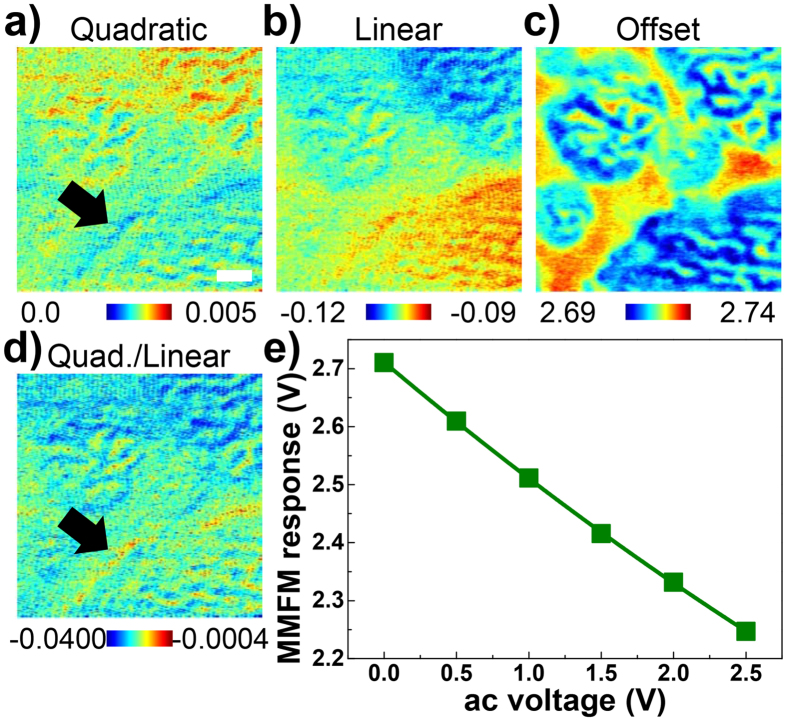
Spatial maps of fitting parameters of the nonlinear equation for the MMFM amplitude images: (**a**) quadratic, (**b**) linear, (**c**) offset coefficients and (**d**) ratio of quadratic and linear coefficients. (**e**) Dots and solid line represent averaged data points and fit, respectively. Scale bar is 0.5 μm.

As mentioned above, since the magnetic forces exerted on the tip associated with the coil and eddy currents are linearly and nonlinearly related with *V*_*ac*_, respectively, we further investigated the details of the MMFM response by fitting with a nonlinear equation. The amplitudes of the MMFM responses in Fig. 5(a) at each pixel were fitted with the following nonlinear equation:


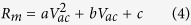


where *R*_*m*_, *V*_*ac*_, *a, b* and *c* represent the MMFM response, amplitude of *V*_*ac*_, quadratic, linear and offset coefficients, respectively. Then, the spatial maps of the quadratic, linear and offset coefficients are extracted as shown in Fig. 6(a–c). In this case, each coefficient stands for the contributions of the eddy and coil currents and those of the static magnetic domains to the MMFM response, respectively.

In the spatial map of the quadratic coefficient in Fig. 6(a), the resin between the particles shows a lower quadratic coefficient than the soft magnetic particles (see the black arrow in Fig. 6(a)). While eddy current is induced within the conductors, it is nearly zero within the resin. This indicates that the eddy current is indeed generated and properly measured by the suggested MMFM technique. However, there might be a very small nonlinear response in the resin, which is expected to be generated by the soft magnetic particles underneath the resin. Interestingly, the stripe patterns of the magnetic domains are also clearly distinguished in the spatial maps of the quadratic and linear coefficients as shown in Fig. 6(a,b). This can be explained by the fact that the contributions of the eddy and coil currents to the MMFM response might be different depending on the pre-existing magnetic domains. The spatial map of the offset coefficient as illustrated in Fig. 6(c) shows the static magnetic domains, which is also represented by the MMFM response with 0 *V*_*ac*_ in Fig. 5(a) (see Supplementary Figure S6).

Figure 6(d) depicts a spatial map of the ratio of the quadratic and linear coefficients, which is meaningful as it shows the overall and relative relationship between the coil and eddy currents. The ratio in the vicinity of the resin is nearly zero (see the black arrow in Fig. 6(d)), whereas the absolute value of the ratio obtained within the soft magnetic particles is relatively larger. Since the eddy current is primarily induced inside the soft magnetic particles, these results can be readily understood. We note that the ratio is negative over the whole area because the direction of the eddy current is opposite that of the coil current.

Finally, a nonlinear relationship, *R*_*m*_ = *0.010V*_*ac*_^2^ *− 0.210V*_*ac*_ + *2.711*, was obtained by fitting the averaged MMFM responses over the entire measured area shown in Fig. 6(e). Since the quadratic coefficient of 0.010 is relatively small compared with others, it is expected that eddy current is barely generated within the commercial MI sample. Nonetheless, we are still able to understand the different behaviors of the static magnetic domains, the eddy and coil currents, and also to estimate the relative contributions from each origin to the MMFM response. We also note that, since the eddy current has a quadratic shape, it can likely be obtained through second harmonic measurements^24^,^25^.

## Conclusion

In summary, we have developed a MMFM technique that allows probing of the multiple magnetic properties associated with eddy current, coil current and magnetic domains. By using the MMFM, we were able to observe local features of magnetic and eddy current responses in the MI. The MMFM images at 0 *V*_*ac*_ show a spatial distribution of the static magnetic domains similar to the images of the conventional MFM. The bias dependent MMFM measurements show larger nonlinear responses, *i.e.* eddy current contribution, inside the soft magnetic particles. Furthermore, analysis of the local magnetic response reveals a clear correlation between the eddy current and pre-existing magnetic domains. This newly proposed method would be very useful for analyzing simultaneous responses from multiple types of magnetic properties such as magnetic and eddy currents and could provide clues about the origin of the loss mechanism in MIs. We further note that the proposed approach for probing magnetic responses including the eddy current can be further extended to analysis of local defects in other material systems because the eddy current is conventionally used to detect local defects in steel or other conducting materials^26^. Hence, this approach can be a suitable tool for probing local magnetic responses as well as local physical features.

## Methods

### Materials

A commercial MI manufactured by Samsung Electro-Mechanics was chosen as a model system. The MI is a thin film type inductor with an inductance of 1 μH and dimensions of 2.08 × 1.7 × 0.98 mm^3^ (width × depth × height). The MI was composed of soft magnetic particles, resin, and a Cu coil (127 μm thick) located in the middle. The soft magnetic particles were composed of amorphous Fe-Cr-Si-B-C compounds with two kinds of powder particles (coarse and fine particles). The coarse and fine particle sizes were 10 ~ 20 and 1.5 ~ 3.5 μm, respectively. The space between the circular magnetic particles was filled with resin. The upper side of the MI was polished for analysis of the magnetic response on the sample surface. The detailed microstructure can be found in supplementary materials (see Supplementary Figure S1).

### Measurements

Ambient AFM studies were performed with a commercial AFM system (NX-10, Park Systems) additionally equipped with a lock in amplifier (SR830, Stanford Research Systems). The MMFM measurements were carried out with a magnetized magnetic tip (Multi75M-G, BudgetSensors). To acquire MFM and MMFM images, the magnetized tip was vibrated at the vicinity of the resonant frequency, 75 kHz, with a lift mode (tip-sample distance: 50 nm). During the operation of MMFM, the frequency of the ac voltage applied to the coil is synchronized with that of the piezo dither using a lock-in amplifier. The sensitivity and calibration coefficients (from *V*_*PSPD*_ in the MMFM image to actual magnetic strength) of the MMFM in this work were roughly estimated as 0.122 μN/Oe and 1.971 Oe/*V*_*PSPD*_, respectively. 1.971 Oe/*V*_*PSPD*_ indicates that 1 V in the MMFM image corresponds to 1.971 Oe (see Supplementary Figure S3 for more details).

## Additional Information

**How to cite this article**: Park, S. *et al.* Probing of multiple magnetic responses in magnetic inductors using atomic force microscopy. *Sci. Rep.* **6**, 20794; doi: 10.1038/srep20794 (2016).

## Supplementary Material

Supplementary Information
