# Supplementary material for: Probing of multiple magnetic responses in magnetic inductors using atomic force microscopy
Source: Sci Rep. 2016 Feb 8;6:20794. doi: 10.1038/srep20794 (PMC4745108; doi:10.1038/srep20794)
Supplement: Supplementary Information [file srep20794-s1.pdf]

Supplementary Information

Probing of multiple magnetic responses in magnetic inductors using atomic force microscopy

Seongjae Park[1,#], Hosung Seo[1,#], Daehee Seol[1], Young-Hwan Yoon,[2] Mi Yang Kim,[2] Yunseok Kim[1]*

[1] School of Advanced Materials Science and Engineering, Sungkyunkwan University (SKKU), Suwon 440-746, Republic of Korea

[2] Fundamental Technology Group, Central R&D Institute, Samsung Electro-Mechanics Co., Suwon 443-743, Republic of Korea

# These authors contributed equally to this work.

[*]Address correspondence to yunseokkim@skku.edu

I. Microstructures of magnetic inductor

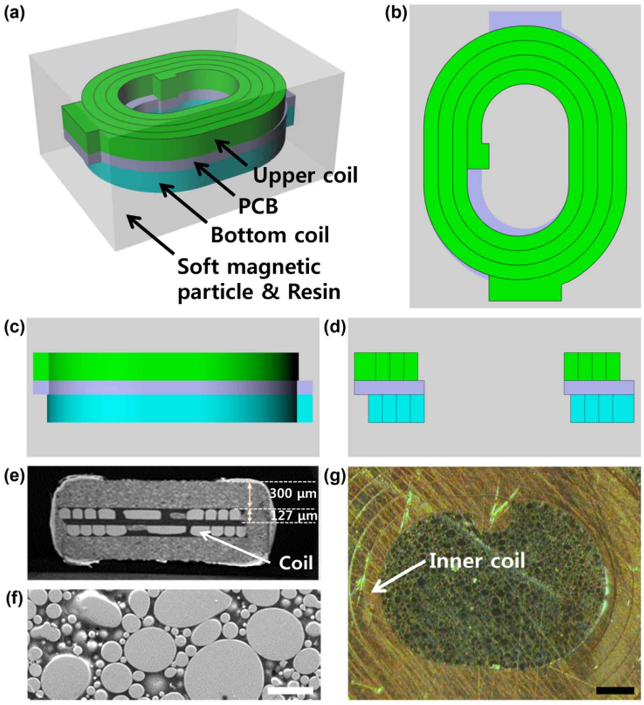

Figure S1. (a) Schematics of the MI with (b) top view, (c) front view and (d) front view of cross-section area at the half. SEM images of (e) cross-sectional and (f) polished surfaces of the MI. (g) OM image of polished surfaces of the MI. White and black scale bars are 20 and 200 μm, respectively. Note that the cross-section of the SEM image shown in Fig. (e) is not exactly cut in half.

Figure S1(a-d) illustrates the schematics of the magnetic inductor (MI) including the top and front views as well as the cross-sectional area for clear understanding of the dimensions of the MI sample. The scanning electron microscopy (SEM) images of cross-sectional and polished surfaces of the MI are also shown in Fig. S1(e) and S1(f). As described in the methods section, the MI was composed of soft magnetic particles and resin, with a Cu coil in the middle. As shown in Fig. S1(g), the coil is wound up with an ellipse shape and the

inner major and minor axes of the coil are 1100 and 810 μm, respectively. (g). The circular particles and the filled space between the particles depict the soft magnetic particles and resin, respectively. The soft magnetic particles are composed of amorphous Fe-Cr-Si-B-C compounds with two kinds of powder particles, which are coarse and fine particles as shown in Fig. S1(f). The coarse and fine particle sizes were 10 ~ 20 and 1.5 ~ 3.5 μm, respectively.

II. Current flow and generated magnetic field through coil in MI

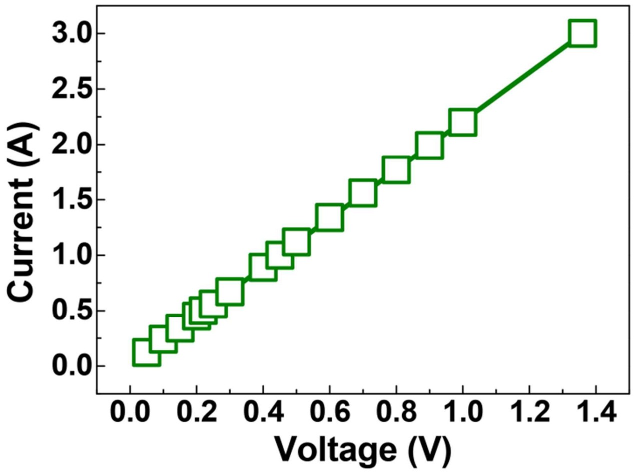

Figure S2. Current as a function of the dc voltage applied to the coil. The square dots and the solid line represent the measured data points and the fit by linear equation, respectively.

The current flow through the coil induces a magnetic field inside the MI. Thus, it is necessary to clarify the relationship between the applied voltage and the current flow in the coil. As the dc voltage applied to the coil increases, the current flow through the coil also increases according to Ohm's law. The linear coefficient obtained from the fit is 2.20, which reflects the 0.46 Ω of resistance across the coil. Thus, 1.00 V_{dc} corresponds to about 2.20 A.

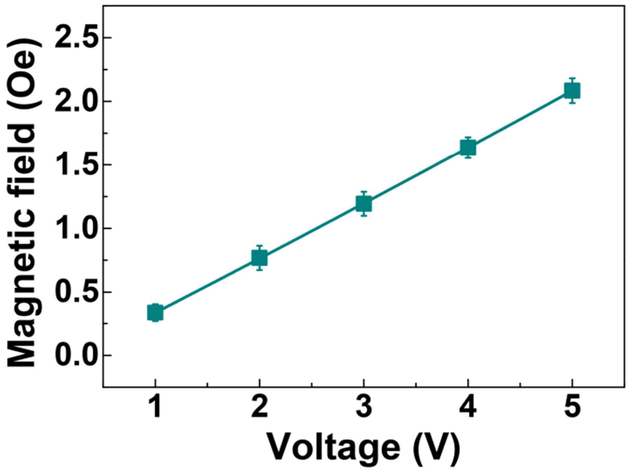

Figure S3. Magnetic field as a function of ac voltage (1 kHz) applied to the coil. The square dots and the solid line represent the measured data points and fit by quadratic equation, respectively. Each value was averaged from 30 measurements.

The magnetic field induced by the coil current was roughly estimated by a commercial guass meter (GM08, Hirst Magnetic Instruments) with a frequency range of 15 to 10 kHz. As the applied voltage to the coil increases, the magnetic field induced by the coil increases as expected. The linear and quadratic coefficients obtained from the fit are 0.414 and -0.078, respectively. Thus 1.00 V_{ac} corresponds to about 0.414 Oe , *i.e.* 0.414 Oe/V_{ac}. We note that, since the magnetic fields were measured by macroscopic tools, these values may not be exactly the same as in the MMFM system.

The sensitivity and calibration coefficient of the MMFM in this work can be roughly obtained from the above measurement combined with the MMFM response as a function of V_{ac} and force-distance measurement. The relationship between the signal detected by a position-sensitive photodiode (PSPD), V_{PSPD}, in the MMFM image and the applied ac voltage V_{ac} can be obtained as 0.210 V_{PSPD}/V_{ac} as shown in Fig. 6. Further, the relation between the force acting on the cantilever and V_{PSPD} can be derived as 1109 nN/V_{PSPD} from force-distance measurements (not shown here) and the spring constant of the cantilever. Thus, these factors

result in a sensitivity of 0.563 μN/Oe, indicating that the magnetic field of 1 Oe exerts the force of 0.563 μN on the cantilever with a calibration coefficient of 1.971 Oe/V_{PSPD}, which indicates that 1 V of the MMFM response corresponds to 1.971 Oe.

III. MMFM images as functions of measuring conditions

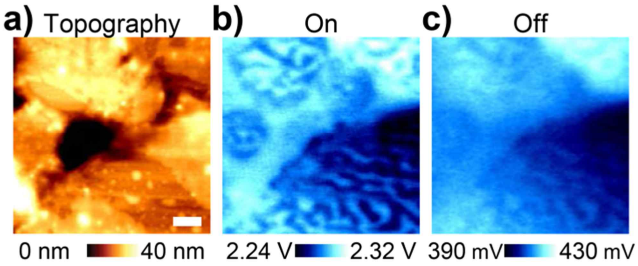

Figure S4. (a) Topography and corresponding (b,c) MMFM amplitude images (b) with and (c) without the use of a piezo dither. Scale bar is 0.5 μm.

The MMFM amplitude images in Figs. 4 and 5 were obtained using a piezo dither synchronized with the ac voltage applied to the coil. However, in principle, the MMFM can be operated without use of the piezo dither. In fact, the contrast of the MMFM amplitude images for both cases can be the same because the magnetic fields induced by coil and eddy currents can solely excite the cantilever. Indeed, even though the response obtained using the piezo dither is much larger, both MMFM amplitude images appear to have identical contrast as shown in Figs. S4(b) and S4(c). The stripe magnetic domain patterns inside the soft magnetic particles are visible in both cases. Nonetheless, in this work, the piezo dither was used for two reasons: 1) the larger response reduces the relative noise level and 2) the conventional MFM can be readily modified to the MMFM set-up because the difference between the conventional MFM and the MMFM is only in the modulation of the input ac voltage.

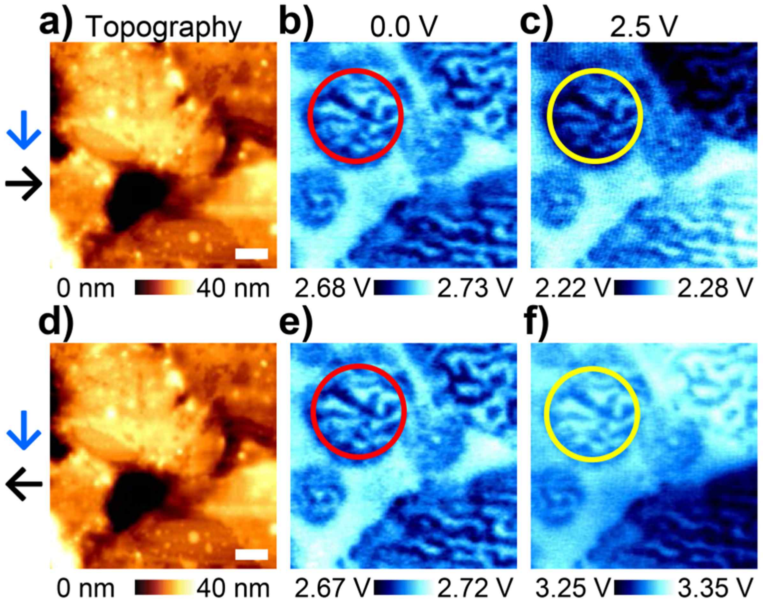

Figure S5. (a,e) Topography and (b-d, f-h) MMFM amplitude images with different directions for the coil current: (a-c): rightward and (d-f) leftward (same as the definition in Fig. 2(c)). The black and blue arrows represent the directions of the coil current and the downward magnetized tip. Each MMFM image was obtained at the applied voltage of (b,e) 0 V_{ac} and (c,f) 2.5 V_{ac}. Scale bar is 0.5 µm.

In addition to the direction of magnetization in the tip, the direction of the coil current can affect the MMFM response with ac voltage. All MMFM responses at 2.5 V_{ac} are either increased or decreased compared to those at 0 V_{ac} depending on the direction of the coil current as shown in Figs. S5(b-c) and S5(e-f). Furthermore, there was no visible change in the contrast of the magnetic domains with respect to ac voltage as mentioned in Fig. 4.

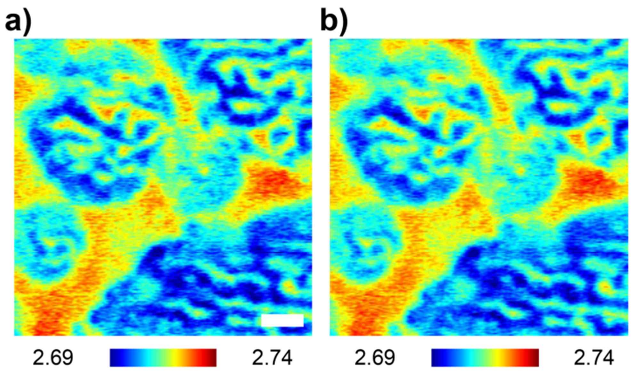

Figure S6. (a) MMFM amplitude image with 0 V_{ac} in Fig. 5(a). and (b) a spatial map of the offset coefficient in Fig. 6(c). Scale bar is 0.5 μm.

The MMFM amplitude image with 0 V_{ac} in Fig. 5(a) is modified to have the same color scale as the spatial map as shown in Fig. S6(a). It is apparent that both images are similar as they depict the same information, *i.e.* static magnetic domains.

IV. EFM analysis under the MMFM setup

In order to analyze the effect of electrostatic contribution to the MMFM response in our experimental setup, the electrostatic response was obtained at the same location as the data shown in Figs. 4 and 5 using electrostatic force microscopy (EFM). Since the experimental setup for obtaining EFM responses is almost the same as that of MMFM, with the exception of using a Pt-coated conductive tip (Multi75E-G, BudgetSensors) instead of a magnetic tip, the obtained EFM response can provide electrostatic contribution to the MMFM response. As shown in Fig. S7, there is no significant difference in the EFM response with respect to V_{ac}. In other words, the electrostatic force exerted between the tip and sample is negligibly small. This might be due to the different pathways for the two ac voltages, which are applied separately to the coil and to the piezo dither to mechanically excite the cantilever.

Alternatively, it may be because the physical distance between the tip and coil is fairly large as shown in Fig. S1.

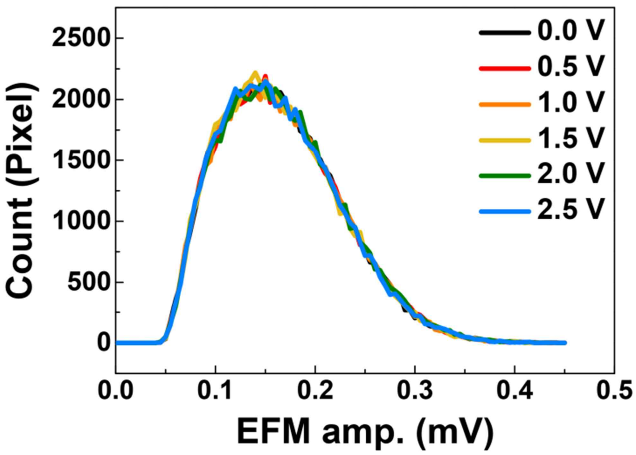

Figure S7. Histograms of EFM amplitudes with respect to V_{ac}.

V. Theoretical calculation of the magnetic forces acting on the tip with different origins

Since there are two magnetic dipoles in our experimental setup, namely the magnetized tip and the magnetic field associated with the sample, coil and eddy currents, the magnetic force can be theoretically calculated with the following assumptions: 1) the magnetized tip is fixed at a constant height with respect to the surface of the sample, and its magnetic moment is also constant, 2) the magnetic field on the sample surface, which results from either the soft magnetic particles in the sample or the coil and eddy currents, is at the center of the circular coil, and the magnetized tip is located along the z axis with respect to the center and 3) the obtained signal contains only the magnetic force exerted along the *z* direction. Then, the force between the two magnetic dipoles, $F_{magnetic}$, acting on the tip can be written as follows

$$F_{magnetic} = \nabla(M_{tip} \cdot B_z) = \frac{\partial(M_{tip} \cdot B_z)}{\partial x}\hat{\imath} + \frac{\partial(M_{tip} \cdot B_z)}{\partial y}\hat{\jmath} + \frac{\partial(M_{tip} \cdot B_z)}{\partial z}\hat{k} \quad \text{(S1)}$$

where M_{tip}, B_z, i, j, and k are the tip magnetization, the magnetic field along the z axis, and the unit vectors along the x, y, and z axes, respectively. The equation can be simplified with assumption 3) and the fact that the angle between the two dipoles is 180 degrees.

$$F_{magnetic} = \frac{\partial(M_{tip} B_z)}{\partial z}\hat{k} \tag{S2}$$

For the magnetic force associated with the sample, eq. S2 becomes a constant with respect to V_{ac} because the magnetic field of the sample is independent of V_{ac} in the present case. Thus, the magnetic force of the sample, F_{sample}, is written as follows

$$F_{sample} = \frac{\partial(M_{tip} \cdot B_{sample})}{\partial z}\hat{k} = F'_{sample} \tag{S3}$$

Note that the term, F', stands for the other terms except V_{ac}. The magnetic field generated by a coil current can be written as follows[1]

$$B_{coil} = \frac{r^2}{2(z^2+r^2)^{\frac{7}{2}}} I = \frac{r^2}{2(z^2+r^2)^{\frac{7}{2}}} \cdot \frac{V_{ac}}{R_{coil}} \tag{S4}$$

Since the current flow through the coil is directly proportional to the applied ac voltage, the term, I, can be transformed into an expression containing the resistance and V_{ac} according to Ohm's law. Then, similar to the magnetic force of the sample, eq. S2 for the magnetic force related to the coil can be rearranged as follows

$$F_{coil} = \frac{\partial(M_{tip} \cdot B_{coil})}{\partial z}\hat{k} = F'_{coil} \cdot V_0 \sin \omega t \tag{S5}$$

The magnetic force for the eddy current can be derived from the equation obtained with the other model system, that is, a moving monopole along the x direction at a constant height from the sample with a specific velocity[2]. Then, the lift and drag forces exerted on the monopole, F_L and F_D , respectively, are theoretically calculated as follows

$$F_L = \frac{\mu_0 q^2}{16\pi {z_0}^2}\left[\frac{1-\omega}{(v^2+\omega^2)^{\frac{1}{2}}}\right], \; F_D = \left(\frac{\omega}{v}\right) F_L \tag{S6}$$

where μ_0, q, z_0, v, and ω are the vacuum permeability, the pole strength, the constant height, the uniform velocity of the moving monopole, and the parameter related to the thin

conducting plate with velocity as the unit, respectively. Then, eq. S6 can be applied in our experimental setup based on the following assumptions: 1) the conducting plate sample and the moving monopole are considered the magnetized tip and the coil following application of ac voltage. 2) The velocity of the moving monopole is regarded as the frequency of the ac voltage. Thus, eq. S6 can be modified with the parameters associated with our model system as follows

$$F_{eddy} = \frac{\mu_0 \mu_r}{16\pi {d_0}^2} f(\omega) {m_{coil}}^2 \quad \text{(S7)}$$

where μ_r, m_{coil}, and d_0 are the relative permeability, the magnetic moment of the coil and the distance between the coil and the tip along the z axis, respectively. Since the magnetic moment of a solenoid is directly proportional to the current flowing through it, eq. S7 can be further arranged as follows

$$F_{eddy} = \frac{\mu_0 \mu_r}{16\pi {d_0}^2} f(\omega)(m'_{coil} \cdot V_0 \sin \omega t)^2 = F'_{eddy} \cdot {V_0}^2(1 - \cos 2\omega t) \quad \text{(S8)}$$

References

1. Kong LS, Chou SY. Quantification of magnetic force microscopy using a micronscale current ring. *Appl Phys Lett* **70**, 2043-2045 (1997).

2. Reitz JR. Forces on Moving Magnets due to Eddy Currents. *Journal of Applied Physics* **41**, 2067-2010 (1970).
